# Supplementary material for: Relationship Between Estimated Drug Distribution of Antiretroviral Therapy and Immune Proteins in Cerebrospinal Fluid During Chronic HIV Suppression
Source: Viruses. 2025 May 23;17(6):749. doi: 10.3390/v17060749 (PMC12197675; doi:10.3390/v17060749)
Supplement: Supplementary file 1 [file viruses-17-00749-s001.zip › viruses-3607169-supplementary.pdf]

Supplementary Table 1. Univariate analysis (linear regressions) for CSF biomarkers

	β (BCa 95%CI), p value (n=275)		
	**CSF CXCL10**	**CSF TNF-α**	**CSF IL-6**
**CPE value**	-0.24 (-0.33; -0.15) <0.001	-0.10 (-0.16; -0.036), 0.002	-0.013 (-0.058; 0.034) 0.571
**Age, years**	0.001 (-0.015; 0.016) 0.898	0.002 (-0.012; 0.015) 0.813	-0.001 (-0.008; 0.005) 0.835
**Male sex, ref. female**	0.38 (0.067; 0.71) 0.017	0.025 (-0.24; 0.32) 0.864	-0.005 (-0.138; 0.132) 0.951
**White race, ref. others**	-0.049 (-0.30; 0.21) 0.690	-0.020 (-0.22; 0.17) 0.843	0.093 (-0.026; 0.216) 0.130
**BMI**	0.009 (-0.015; 0.033) 0.416	-0.003 (-0.21; 0.015) 0.772	-0.002 (-0.014; 0.010) 0.696
**HCV+ serostatus, ref. negative**	-0.16 (-0.43; 0.13) 0.292	-0.12 (-0.33; 0.12) 0.251	-0.082 (-0.159; 0.362) 0.386
**Estimated duration of HIV infection, years**	0.25 (-0.044; 0.56) 0.110	0.12 (-0.12; 0.35) 0.311	0.093 (-0.134; 0.356) 0.445
**Current CD4+ T cell count, cells/μL**	-0.44 (-0.91; 0.004) 0.067	0.018 (-0.28; 0.25) 0.907	0.020 (-0.159; 0.184) 0.791
**CD4/CD8 ratio**	-0.47 (-0.92; -0.027) 0.034	-0.018 (-0.33; 0.30) 0.898	0.025 (-0.062; 0.043) 0.172
**Nadir CD4+ T cell count, cells/μL**	-0.21 (-0.41; -0.014) 0.032	-0.040 (-0.19; 0.11) 0.590	-0.040 (-0.140; 0.056) 0.413
**AIDS episode, ref. none**	0.28 (0.021; 0.55) 0.037	0.21 (0.036; 0.39) 0.026	0.063 (-0.068; 0.176) 0.300
**PI use, ref. no use**	0.38 (0.15; 0.59) 0.005	0.13 (-0.049; 0.33) 0.155	0.076 (-0.042; 0.209) 0.210
**NNRTI use, ref. no use**	-0.30 (-0.55; -0.053) 0.025	-0.081 (-0.28; 0.092) 0.392	-0.032 (-0.153; 0.082) 0.594
**Duration of the current ART regimen, months**	-0.47 (-0.70; -0.20) <0.001	-0.29 (-0.47; -0.11) 0.002	-0.001 (-0.004; 0.002) 0.423
**Duration of all ART regimens, months**	-0.29 (-0.69; 0.022) 0.177	-0.12 (-0.30; 0.077) 0.219	-0.048 (-0.136; 0.034) 0.263

Legend: β= beta coefficient; BCa, Bias corrected and accelerated; 95%CI, 95% confidence interval; CPE= Central Nervous System Penetration Efficacy; CXCL10 = C-X-C Motif Chemokine Ligand 10; CSF= cerebrospinal fluid; TNF-α= Tumor Necrosis Factor alpha; IL-6, interleukin 6; BMI, body mass index; HCV, hepatitis C virus; PI= protease inhibitor; NNRTI= non-nucleoside reverse transcriptase inhibitor; ART= combination antiretroviral therapy.

Supplementary Table 2. Univariate analysis (linear regressions) for CSF biomarkers and single antiretrovirals grouped by CPE ranking

	CSF CXCL10		CSF TNF-α		CSF IL-6	
	β (BCa 95%CI), p	D	β (BCa 95%CI), p	D	β (BCa 95%CI), p	D
	**Low CPE (≤2)**					
**TDF use (CPE 1), ref. no use**	0.46 (0.21; 0.72) 0.002	↑	0.18 (0.009; 0.36) 0.045	↑	0.070 (-0.010; 0.14) 0.080	-
**ATV/r use (CPE 2), ref. no use**	0.57 (0.30; 0.85) <0.001	↑	0.39 (0.15; 0.66) 0.003	↑	-0.020 (-0.11; 0.073) 0.642	-
**3TC use (CPE 2), ref. no use**	-0.24 (-0.54; 0.087) 0.137	-	-0.099 (-0.35; 0.16) 0.444	-	-0.045 (-0.13; 0.022) 0.247	-
	**Moderate CPE (=3)**					
**LPV/r use (CPE 3), ref. no use**	-0.14 (-0.46; 0.22) 0.407	-	-0.29 (-0.48; -0.072) 0.009	↓	-0.070 (-0.15; 0.016) 0.111	-
**EFV use (CPE 3), ref. no use**	-0.20 (-0.48; 0.063) 0.152	-	-0.029 (-0.23; 0.16) 0.763	-	0.037 (-0.051; 0.15) 0.455	-
**FTC use (CPE 3), ref. no use**	0.076 (-0.20; 0.35) 0.567	-	0.12 (-0.077; 0.33) 0.246	-	0.015 (-0.071; 0.10) 0.741	-
**ABV use (CPE 3), ref. no use**	-0.096 (-0.48; 0.28) 0.611	-	-0.007 (-0.23; 0.21) 0.953	-	0.003 (-0.084; 0.098) 0.957	-
	**High CPE (=4)**					
**NVP use (CPE 4), ref. no use**	-0.33 (-0.59; -0.073) 0.012	↓	-0.19 (-0.37; -0.019) 0.032	↓	0.025 (-0.81; 0.14) 0.667	-
**ZDV use (CPE 4), ref. no use**	-0.56 (-0.84; -0.25) 0.003	↓	-0.26 (-046; -0.078) 0.012	↓	-0.035 (-012; 0.058) 0.427	-

Legend: β= beta coefficient; BCa, Bias corrected and accelerated; 95%CI, 95% confidence interval; D, direction of biomarker levels in case of antiretroviral use (↑, higher; ↓ lower); CXCL10 = C-X-C Motif Chemokine Ligand 10; CSF= cerebrospinal fluid; TNF-α= Tumor Necrosis Factor alpha; IL-6, interleukin 6; TDF, tenofovir disoproxil; ATV/r, atazanavir boosted with ritonavir; 3TC, lamivudine; LPV/r, lopinavir boosted with ritonavir; EFV, efavirenz; FTC, emtricitabine; ABV, abacavir; NVP, nevirapine; ZDV, zidovudine.

Supplementary Table 3. Multivariable models for CSF CXCL10 including single antiretrovirals.

	CSF CXCL10 (n=275)			
	**Model 1**		**Model 2**	
	**R^2=0.16, p<0.001 aβ (BCa 95%CI), p**	**R^2=0.15, p<0.001* aβ (BCa 95%CI), p**	**R^2=0.15, p<0.001 aβ (95%CI), p**	**R^2=0.14, p<0.001* aβ (95%CI), p**
**CPE value**	-0.17 (-0.34; -0.002), p=0.048	-*	-0.16 (-0.26; -0.051) p=0.003	-*
**Age, years**	-	-	0.008 (-0.008; 0.023), p=0.327	0.008 (-0.007; 0.023), p=0.290
**Male sex, ref. female**	0.35 (0.030; 0.67), p=0.030	0.36 (0.046; 0.66), p=0.022	0.36 (0.043; 0.68), p=0.026	0.33 (0.016; 0.65), p=0.040
**White race, ref. others**	-	-	0.003 (-0.25; 0.26), p=0.981	-0.019 (-0.28; 0.24), p=0.887
**CD4/CD8 ratio**	-0.032 (-0.50; 0.43), p=0.892	-0.042 (-0.50; 0.47), p=0.850	Excluded	Excluded
**Nadir CD4+ T cell count, cells/μL**	-0.14 (-0.41; 0.14). p=0.322	-0.12 (-0.39; 0.14), p=0.385	-0.18 (-0.39; 0.019), p=0.076	-0.19 (-0.39; 0.014), p=0.068
**AIDS episode, ref. none**	0.13 (-0.19; 0.46), p=0.423	0.13 (-0.19; 0.44), p=0.390	Excluded	Excluded
**PI use, ref. no use**	0.20 (-0.41; 0.82), p=0.512	0.21 (-0.35; 0.84), p=0.437	Excluded	Excluded
**NNRTI use, ref. no use**	0.23 (-0.39; 0.85), p=0.468	0.096 (-0.51; 0.67), p=0.724	Excluded	Excluded
**Duration of the current ART regimen, months**	-0.34 (-0.60; -0.074), p=0.013	-0.32 (-0.61; -0.061), p=0.022	-0.36 (-0.60; -0.11), p=0.004	-0.36 (-0.61; -0.11), p=0.005
**NVP use, ref. no use**	0.056 (-0.29; 0.40), p=0.749	0.10 (-0.25; 0.48), p=0.575	Excluded	Excluded
**ZDV use, ref. no use**	0.16 (-0.35; 0.68), p=0.534	-0.12 (-0.56; 0.35), p=0.573	Excluded	Excluded
**ATV/r use, ref. no use**	0.27 (-0.10; 0.65), p=0.153	0.35 (-0.026; 0.74), p=0.073	0.29 (-0.20; 0.59), p=0.066	0.45 (0.17; 0.73), p=0.002
**TDF use, ref. no use**	0.12 (-0.29; 0.53), p=0.569	0.30 (-0.11; 0.72), p=0.151	Excluded	0.29 (0.022; 0.56), p=0.034

*CPE was not included in these models. Models 1 were performed including all covariates with significant associations at univariable analysis (p<0.05) and entry method; models 1 underwent bootstrapping in 1,000 samples (the shown aβ, 95%CI, and p values are bias corrected and accelerated). Models 2 were performed including all covariates with significant associations at univariable analysis (p<0.05) and age, sex, race, and duration of ART regardless of univariable association; then backward selection was applied based on the Akaike information criterion. Variables labeled as "Excluded" were entered into the model but not retained after backward selection. As such, beta coefficients and p-values are not reported. Legend: aβ= adjusted beta coefficient; BCa, Bias corrected and accelerated; 95%CI, 95% confidence interval; CPE= Central Nervous System Penetration Efficacy; CXCL10 = C-X-C Motif Chemokine Ligand 10; CSF= cerebrospinal fluid; PI= protease inhibitor; NNRTI= non-nucleoside reverse transcriptase inhibitor; ART= combination antiretroviral therapy; NVP, nevirapine; ZDV, zidovudine; ATV/r, atazanavir boosted by ritonavir; TDF, tenofovir disoproxil.

Supplementary Table 4. Multivariable models for CSF TNF-α including single antiretrovirals.

	**CSF TNF-α (n=275)**		
	**Model 1**		**Model 2°**
	**R^2=0.11, p<0.001 aβ (BCa 95%CI), p**	**R^2=0.10, p<0.001* aβ (BCa 95%CI), p**	**R^2=0.09, p<0.001 aβ (BCa 95%CI), p**
**CPE value**	-0.03 (-0.14; -0.078), p=0.558	-*	Excluded
**Age, years**	-	-	0.004 (-0.007; 0.016), p=0.448
**Male sex, ref. female**	-	-	-0.007 (-0.25; 0.23), p=0.956
**White race, ref. others**	-	-	-0.032 (-0.22; 0.16), p=0.739
**AIDS episode, ref. none**	0.17 (-0.020; 0.37), p=0.079	0.18 (-0.005; 0.35), p=0.058	Excluded
**Duration of the current ART regimen, months**	-0.23 (-0.42; -0.049), p=0.013	-0.23 (-0.41; -0.037), p=0.019	-0.27 (-0.45; -0.090), p=0.004
**NVP use, ref. no use**	-0.011 (-0.35; 0.37), p=0.951	-0.095 (-0.33; 0.14), p=0.414	Excluded
**ZDV use, ref. no use**	0.16 (-0.35; 0.68), p=0.534	-0.042 (-0.31; 0.22), p=0.766	Excluded
**ATV/r use, ref. no use**	0.22 (-0.020; 0.47), p=0.071	0.26 (0.025; 0.52), p=0.039	0.35 (0.14; 0.56), p=0.001
**TDF use, ref. no use**	-0.030 (-0.33; 0.27), p=0.846	0.004 (-0.22; 0.23), p=0.975	Excluded
**LPV/r use, ref. no use**	-0.26 (-0.55; 0.027), p=0.076	-0.25 (-0.46; -0.041), p=0.023	Excluded

*CPE was not included in these models. °Backward selection excluded CPE from the final model that included it, therefore both models 2 (including and not including CPE) resulted identical. Models 1 were performed including all covariates with significant associations at univariable analysis (p<0.05) and entry method; models 1 underwent bootstrapping in 1,000 samples (the shown aβ, 95%CI, and p values are bias corrected and accelerated). Models 2 were performed including all covariates with significant associations at univariable analysis (p<0.05) and age, sex, race, and duration of ART regardless of univariable association; then backward selection was applied based on the Akaike information criterion. Variables labeled as "Excluded" were entered into the model but not retained after backward selection. As such, beta coefficients and p-values are not reported. Legend: aβ= adjusted beta coefficient; BCa, Bias corrected and accelerated; 95%CI, 95% confidence interval; CPE= Central Nervous System Penetration Efficacy; CSF= cerebrospinal fluid; TNF-α= Tumor Necrosis Factor alpha; ART= combination antiretroviral therapy; NVP, nevirapine; ZDV, zidovudine; ATV/r, atazanavir boosted by ritonavir; TDF, tenofovir disoproxil; LPV/r, lopinavir boosted by ritonavir.
